# The Effect of Sedation During Upper Gastrointestinal Endoscopy

**DOI:** 10.4103/1319-3767.70616

**Published:** 2010-10

**Authors:** Atul Sachdeva, Ashish Bhalla, Ashwani Sood, Ajay Duseja, Vijay Gupta

**Affiliations:** Department of Internal Medicine, Government Medical College and Hospital, Chandigarh, India; 1 Post-Graduate Institute of Medical Education and Research, Chandigarh, India

**Keywords:** Endoscopic procedures, gastrointestinal endoscopy, patient’s perception, sedation

## Abstract

**Background/Aim:**

We aimed to study whether sedation reduces discomfort during endoscopy and a comparison of longer-acting diazepam with shorter-acting midazolam.

**Patients and Methods:**

A prospective, randomized, single-blinded study was conducted at the Department of Medicine at Government Medical College and Hospital, Chandigarh, and was completed over a period of 6 months. The patients were randomized to receive either placebo or sedation with midazolam or diazepam before endoscopy. The endoscopist and the observer recording patient’s/physician’s responses were blinded to the drugs administered. Two hundred and fifty two consecutive patients undergoing diagnostic or therapeutic upper gastrointestinal endoscopy were recruited. The patient’s discomfort and the physician’s comfort during the procedure were recorded on a visual analogue scale rated from 1-10 with-in 10 minutes of the procedure by an independent observer. The Patient’s discomfort ratings were further divided into 3 groups, comfortable (score, 1-3), satisfactory (score, 4-7) and uncomfortable (a score of >7). Similarly the physician’s ease of performing the procedure was also recorded on the same scale. This was again divided into 3 groups: easy (score, 1-3), satisfactory (score, 4-7) and difficult (a score of >7).

**Results:**

Out of the total of 252 patients, 82 patients received no sedation (group I), 85 received diazepam (group II) and 85 received midazolam (group III). There was no statistical difference in the discomfort experienced by the patients during endoscopy when sedation was used (*P*=0.0754). Out of 252 patients, 49 underwent endoscopic procedures. Nineteen patients were included in group I, 18 in group II and 12 in group III. Only 10 (20%) patients undergoing endoscopic procedures complained of significant discomfort, but there was no difference in the ones undergoing interventions with or without sedation (*P*=0.854). The physicians were more comfortable in performing endoscopic procedure in sedated patients, however, the difference between patients in group II and group III was not statistically significant (*P*=0.0461). Both diazepam and midazolam fared equally well in increasing physician’s comfort (*P*=0.617).

**Conclusion:**

There was no difference in the patient’s discomfort with regard to the sedative used (midazolam or diazepam). Although endoscopy was easy or satisfactory in the majority of patients in the unsedated as well as the sedated groups, more often the endoscopist found it difficult to do endoscopy on the unsedated patients.

Upper gastrointestinal endoscopy is a widely used procedure for diagnosis and treatment of upper gastrointestinal diseases. Although it is considered a safe and well-tolerated procedure, significant discomfort has been noted in patients undergoing endoscopy without sedation. It is also known that endoscopists tend to underestimate the discomfort of patients.[1] The fiber-optic endoscopes have improved the quality and safety of the procedure, and the focus now is to reduce the discomfort experienced by the patients during the procedure. Recent studies have documented that although sedated diagnostic endoscopy is costlier, yet it increases the rate of successful endoscopies[2] and makes the procedure more tolerable.[3,4] Most endoscopists prefer sedation during the procedure, but of late there has been a debate on the use of sedatives and their effects on patients undergoing procedures. The introduction of anesthetic agents in the endoscopy suite has also generated a debate on “Who should be administering these agents?” Since sedatives are cheaper and safer alternatives to anesthetic agents and can be administered by any qualified physician (not necessarily an anesthetist), our idea of performing this study was to look for an agent that would reduce both patient’s and physician’s discomfort in the endoscopy suite.

The aim of this study was to investigate the effect of sedation on the discomfort felt by the patients undergoing upper gastrointestinal endoscopy for diagnostic or therapeutic purpose. We also looked at the ease of performing the procedure under sedation from the physician’s point of view. We also compared the following two agents with regard to their effect on the physician’s and patient’s comfort during the procedure; diazepam, a long-acting benzodiazepine, and midazolam, a shorter acting agent.

## PATIENTS AND METHODS

### Place of study

The study was conducted at the Department of Medicine, Government Medical College and Hospital Chandigarh, and was completed over a period of 6 months. The study was approved by the institute’s ethics committee.

### Study design

This was a prospective, randomized, single-blinded study. The patients were randomized to receive either placebo or sedation with midazolam or diazepam before the procedure. The sample size was calculated in consultation with the statistician. The randomization of the patients into three groups was done in consultation with a statistician, and the method followed was of block randomization. The endoscopist and the investigator recording the data were not aware of the nature of medication given to the patients, as it was administered by a technician. The code was broken for analysis of the data collected during the procedure.

### Study population

We screened 356 patients and selected 252 consecutive patients undergoing diagnostic or therapeutic upper gastrointestinal endoscopy. The eligibility criteria included patients of all age groups and absence of history of intolerance to benzodiazepines. Patients with history of uncontrolled pulmonary or cardiac disease, concomitant treatment with psychotropic drugs like benzodiazepines, and pregnant women were excluded. Fifty four patients did not give consent for participation in the study, so they were excluded. The written, explained consent was obtained before the procedure was started.

Group I received no sedation but received only intravenous saline (placebo group). Group II received pre-endoscopic, conscious sedation with diazepam and group III received pre-endoscopic conscious sedation with midazolam. No local anesthetics were used during the procedure.

Sedation was given intravenously, and the dosage used was 5 mg of diazepam or 5 mg of midazolam. The dose of midazolam was reduced to 3.0 mg in patients >65 years of age. The sedation was administered once the patient and the physician were both ready for the procedure. Just before beginning the procedure, the intravenous injection was given by a technician, without knowledge to the drug administered.

The endoscopies were done in the left lateral position, by two experienced endoscopists, using Olympus GIF type XQ30 endoscope (Olympus Optical Co. Ltd., Tokyo, Japan), with an outer diameter of 9.8 mm. Endoscopic procedures like biopsies, sclerotherapy, and dilatations were performed wherever indicated.

During and after the procedure, peripheral oxygen saturation was monitored with a finger probe using a portable pulse oximeter. Heart rate and blood pressure were monitored with a Lohmeier 608 multi-channel monitor (made in Germany) before, during and up to 15 minutes after the procedures. Monitoring was extended up to 30 minutes post-procedure in elderly patients and patients having abnormal readings.

### Data recording

The patient’s discomfort and the physician’s comfort during the procedure were recorded on a visual analogue scale rated from 1 to 10. The patients were explained about the visual analogue scale and the ratings, by an independent investigator, before being taken into the endoscopy suite. The score of 1 was given to the minimum level of discomfort and 10 to the maximum level of discomfort. The patient was asked to rate the discomfort experienced by him/her during the procedure, after he/ she was shifted to the bed from the endoscopy table (within 10 minutes of the procedure) for observation.

The rating was then noted by the investigator, within 10 minutes of completion of the procedure. Based on the readings, the responses were further divided into three groups, comfortable (score, 1-3), satisfactory (score, 4-7) and uncomfortable (score, >7). Similarly the physician’s ease of performing the procedure was also recorded on the same scale, after the completion of the procedure. The responses were further categorized into three groups; easy (score, 1-3), satisfactory (score, 4-7) and difficult (score, >7). The data was analyzed using SPSS statistical software, and the variables were analyzed using the chi-square test. The results are presented as a percentage of responses obtained. This is a post hoc analysis of the study designed to look at the cardiorespiratory compromise during endoscopy and the effect of sedation on it.

### Observations

A total of 356 patients were screened, of them, 252 patients were included in the study. 143 patients were male and 109 were female, with an average age of 41.18 ± 15.54 years. Eighty two patients received no sedation (group I), 85 received diazepam (group II) and 85 received midazolam (group III). The demographic and clinical characteristics of the patients in the three study groups were similar [Table 1].

**Table 1:** Baseline characteristics of all patients

Baseline characteristics	Group I (placebo)	Group II (diazepam)	Group III (midazolam)
n	82	85	85
Age (in years)	41.70 ± 15.36	39.91 ± 16.10	41.98 ± 15.26
Sex-male	45	50	48
Female	37	35	37
Weight	57.13 ± 12.61	56.85 ± 12.69	58.44 ± 10.61
Endoscopy duration (minutes)	3.0 ± 1.58	3.29 ± 1.83	3.19 ± 1.19
Procedure done			
Biopsies	14	15	9
Dilations	3	0	2
Endoscopic sclerotherapy	2	3	1

Endoscopy was performed successfully in all the patients. A total of 49 therapeutic or diagnostic interventions, viz, 38 biopsies, 5 esophageal dilations and 6 sclerotherapies of esophageal varices, were undertaken during endoscopy [Table 1]. Mean duration of endoscopy was 3.0±1.58 minutes in group I, 3.29 ± 1.85 minutes in group II, and 3.19 ± 1.19 minutes in group III. In groups I and II, the number of interventions undertaken was similar (19 and 18, respectively); however, it was less in group III (12 only).

Discomfort was experienced during endoscopy in 4.87% of patients in the placebo group, and was similar in the other two groups in whom sedation was used. As many as 3.5% patients experienced discomfort during endoscopy when either diazepam or midazolam was used as pre-medication. (*P*=0.765) [Table 2]. Although there was a trend towards lesser discomfort when sedation was used, this difference was not statistically significant when compared to the discomfort with the use of placebo. The percentages of patients feeling comfortable during endoscopy did not differ much in all the three groups (46%, 56% and 52%, respectively, in groups I, II and III). When we looked at the patient’s comfort levels in the cohort of patients undergoing any endoscopic intervention, only 31.57% were comfortable in the placebo group, whereas, 55% patients were comfortable when diazepam was given as pre-medication and 66.66% felt comfortable when midazolam was used. (*P*=0.1277). The percentage of patients feeling uncomfortable during the procedures, was however similar, 21%, 17% and 25% in groups I, II and III, respectively [Table 3].

**Table 2:** Patient’s comfort and physician’s ease in performing endoscopy

	Group I (placebo)	Group II (diazepam	Group III (midazolam)	*P* value
n	82	85	85	
Duration (minutes)	3.0 ± 1.58	3.29 ± 1.83	3.19 ± 1.19	
Patient’s comfort				
Comfortable	38 (46.34)	46 (56.47)	45 (52.94)	0.557
Satisfactory	40 (48.78)	36 (47.35)	37 (43.52)	0.674
Uncomfortable	4 (4.87)	3 (3.52)	3 (3.5)	0.876
Physician’s ease				
Easy	42 (57.21)	48 (56.47)	44 (51.76)	0.754
Satisfactory	32 (39.02)	34 (40)	38 (44.75)	0.725
Difficult	8 (9.75)	3 (3.5)	3 (3.5)	0.129

Figures in parentheses are in percentage

**Table 3:** Patient’s comfort and physician’s ease in performing endoscopy in patients undergoing interventions

	Group I (placebo)	Group II (diazepam	Group III (midazolam)	*P* value
n	19	18	12	
Duration (minutes)	3.19 ± 1.8	3.24 ± 1.65	3.26 ± 1.1	
Patient’s comfort				
Comfortable	6 (31.57)	10 (55.55)	8 (66.66)	0.127
Satisfactory	9 (47.36)	5 (27.77)	1 (8.33)	0.067
Uncomfortable	4 (21.05)	3 (16.7)	3 (25)	0.854
Physician’s ease				
Easy	4 (21.05)	11 (61.11)	6 (50)	0.041
Satisfactory	9 (47.36)	6 (33.33)	4 (33.33)	0.617
Difficult	6 (31.57)	1 (5.5)	2 (16.66)	0.121

Figures in parentheses are in percentage

When we looked at the physician’s comfort levels while performing endoscopy, we observed that 57% of the times physicians in group I, 56% of the times physicians in group II and 52% of the times physicians in group III felt that performing endoscopy was easy; but physicians found it more difficult to perform endoscopy in non-sedated patients (9.7% in the placebo group *vs*. 3.5% each in the sedation groups). [Table 2]. The physician’s discomfort was even more evident in the patients where endoscopic interventions were done. Only in 21% of cases, the physicians found endoscopic interventions easy in patients who were nonsedated. In groups II and III, physicians were able to perform interventions easily in 61% and 50% of cases, respectively. (*P*=0.0461) [Table 3]. Similarly in nonsedated patients, endoscopic interventions were considered difficult in as many as 31% of cases by the physicians; whereas in patients given pre-medication, this number was 5.5% and 16.6%, respectively, in groups II and III (*P*=0.1225) [Table 3].

When we compared diazepam and midazolam, there was no significant difference in the patient’s perception of discomfort and the physician’s perception of comfort during endoscopy in patients. A higher number of patients (66.7%), given midazolam, felt comfortable during endoscopic interventions as compared to those given diazepam (55%), but more physician’s felt comfortable with diazepam (61%) than midazolam (50%) while carrying out the procedures [Table 3]. This difference, however, did not reach statistical significance (*P*=0.34).

## DISCUSSION

It is a well-known fact that a significant level of discomfort is felt by patients undergoing endoscopy without sedation, and there is a significant underestimation of patients’ discomfort by the endoscopists.[1] However, there have been concerns regarding the safety and efficacy of sedative use in patients undergoing endoscopy.[1,2,4]

The aim of using sedation during endoscopy, is to increase the patient’s satisfaction, shorten duration of the procedure and to make the procedure safer. Endoscopists have used alternatives to pharmacological sedation, such as relaxing music, and using small-caliber endoscopes for unsedated per-oral gastroscopy. Some have resorted to using magnetic endoscopic imaging to increase tolerance and reduce discomfort.[5] However, imaging cannot entirely substitute endoscopes. Imaging can help in diagnosis, but the endoscopic procedures have to be performed under direct vision.

Local anesthetic sprays have been used effectively, and some authors feel that their use should be encouraged as they are safer agents.[6] Since we had not used local anesthetics in any case in our study group, we cannot comment on the safety and efficacy of the same.

Benzodiazepines have been widely used and can be safely administered without the presence of a qualified anesthetist, however, effects of diazepam can last for a long period, resulting in post-procedure sedation, and the effect of midazolam on post-procedural amnesia is well known.[5,7]

Midazolam is the most commonly used sedative during endoscopic procedures, and its use in the dose of 0.06 mg/kg is associated with maximum effect and least amnesia. Amnesia increases when dosage of midazolam is increased to 0.09 mg/kg.[8]

We used a dose of 5 mg of midazolam, which is slightly on the higher side of the recommended dose, but we did not observe any significant paradoxical response or amnesia in any of our patients. All our patients were able to indicate the degree of discomfort, however, an error in judgment of discomfort during the stage of conscious sedation cannot be entirely ruled out.

In a recent double-blind, placebo-controlled trial in Canadian ambulatory adult population, it was noted that sedation resulted in more successful endoscopies, and the authors concluded that sedation is an effective strategy in increasing the rate of successful endoscopies, as well as the level of patient’s satisfaction, and willingness to repeat procedures.[2] In our study, the patient’s level of discomfort was comparable in the sedated and nonsedated states, but more patients reported comfort during endoscopic interventions when sedated. This suggests that sedation may be slightly better than no sedation in a subgroup where endoscopic interventions are planned/indicated.

Studies have shown that conscious sedation by using IV midazolam can improve the tolerance to endoscopy,[3,4] and addition of opiates to midazolam adds no benefit from the patient’s viewpoint, whereas endoscopists were found to be more comfortable when both medications were used together.[9] Our observations are similar; there was no difference in the patient’s level of discomfort with sedation. Even though the endoscopy was easy or satisfactory in the majority of patients in the unsedated and sedated groups, the endoscopists found it difficult to perform endoscopy in unsedated patients more often (9.75% *vs*. 3.5%), especially if procedures were to be performed. There was no difference observed in the efficacy of diazepam *vs*. midazolam used for sedation.

Although both the drugs fared equally well in our study, earlier studies have reported midazolam to be a better agent than diazepam. Amnesia with regards to the procedure may be a contributing factor in reducing patient’s perception of discomfort.[10]

The trends world over have changed regarding the use of sedation during endoscopy with the arrival of propofol on the scene.[11–13] Intravenous propofol, either alone or with concomitant benzodiazepines or opioids, has generated widespread interest. The quality of sedation is better and recovery time is shorter,[13] but with a narrow therapeutic range.[14] A recent meta-analysis suggests that propofol is as safe as midazolam.[15] Since we did not include this drug in our study, we cannot compare its safety and efficacy with midazolam. The sedation achieved may be better with propofol; however, the need of having an anesthetist for endoscopy and the fact that significant cardiorespiratory compromise may develop in a small number of patients, has resulted in apprehensions regarding use of this agent during endoscopy at our center.

We have shown earlier that both midazolam and diazepam are not associated with significant hemodynamic compromise in the dosage used and can be safely administered during endoscopy.[16] Although sedation with midazolam or diazepam may not have reduced patient’s discomfort during endoscopy significantly, yet there was a trend towards increased physicians’ comfort level in performing endoscopies, especially when procedures were needed.

## CONCLUSIONS

We can conclude that although sedation with benzodiazepines may not result in significant reduction in discomfort experienced by patients, yet their use makes physicians feel comfortable when used prior to performing endoscopic procedures. Since the number of patients in both the groups was very small and was further reduced when patients were subgrouped into the ones undergoing procedures, a prospective large randomized study would be needed to resolve the unanswered issues.
